# The Invisible Carbon Footprint as a hidden impact of peatland degradation inducing marine carbonate dissolution in Sumatra, Indonesia

**DOI:** 10.1038/s41598-018-35769-7

**Published:** 2018-11-27

**Authors:** Francisca Wit, Tim Rixen, Antje Baum, Widodo S. Pranowo, Andreas A. Hutahaean

**Affiliations:** 1 Leibniz Center for Tropical Marine Research (ZMT), Fahrenheitstrasse 6, 28359 Bremen, Germany; 2 Institute of Geology, University of Hamburg, Bundesstrasse 55, 20146 Hamburg, Germany; 3 Research & Development Center for Marine & Coastal Resources (P3SDLP), Gedung II BALITBANGKP, Jalan Pasir Putih II, Ancol Timur, Jakarta, 14430 Indonesia; 4 Coordinating Ministry of Maritime Affairs, Jalan. MH. Thamrin No. 8, Jakarta, 10340 Indonesia

## Abstract

In Indonesia, land use change (LUC) in the form of peatland degradation induces carbon loss through direct CO_2_ emissions, but also via soil leaching of which circa 50% is decomposed and emitted as CO_2_ from the rivers. However, the fate of the remaining exported leached carbon is uncertain. Here, we show that the majority of this carbon is respired in the estuaries and emitted to the atmosphere. However, a portion is adsorbed into the marine carbon pool where it favors CaCO_3_ dissolution and can therefore be seen as the invisible carbon footprint. We conclude that the effects of LUC stretch beyond the terrestrial realm and are not limited to CO_2_ emissions, but also affect marine ecosystems. Considering the ecological and economical importance of these ecosystems, it is important that this so far invisible carbon footprint, as well as the aquatic and marine CO_2_ emissions, are included in climate mitigation strategies.

## Introduction

Peatland degradation in Southeast Asia is recognized as an important carbon source to the atmosphere^1^ albeit so far not yet considered in all global estimates of CO_2_ emissions. It would increase the LUC emissions from 1100 Tg C yr^−1^ (ref.^2^) to 1389 ± 938 Tg C yr^−1^ (26%) only by considering CO_2_ emissions caused by peat oxidation and forest fires (289 ± 138 Tg C yr^−1^) (ref.^3^). In Indonesia, regrowth of secondary vegetation could reduce this CO_2_ emission to 105 Tg C yr^−1^ (ref.^4^). However, this estimate would again enhance by 42% to 149 Tg C yr^−1^, as a recent study^5^ revealed that degradation of peatlands has increased carbon leaching from soils by 200% with a leaching rate of 183 g C m^−2^ yr^−1^, as opposed to 62 g C m^−2^ yr^−1^ from pristine peatlands^6^. This increase results partly from changes in the hydrological cycle due to drainage (38%), but is primarily due to regrowth of secondary vegetation (62%) with leaves consisting of relatively labile organic carbon. Despite the carbon-enriched peat soils and enhanced leaching rate, the location of the peatlands near the coast limits the decomposition of leached carbon in the rivers by reducing its residence time in the river, which leads to a relatively modest river outgassing rate of 21–25 Tg C yr^−1^ (87–109 g C m^−2^ yr^−1^) in Indonesian disturbed peatlands^5,7^. With roughly half of the carbon that enters the freshwater system being decomposed and emitted into the atmosphere, it remains unclear what the fate of the exported riverine carbon is once it has reached the estuaries and coastal ocean. In general, tropical estuaries and coastal oceans are heterotrophic systems emitting CO_2_^8–10^. In contrast, a recent study^11^ shows that coastal oceans in west Southeast Asia are considered to be a carbon sink, which would imply that the carbon that is leached from peat soils and exported via the rivers to the coastal ocean is absorbed in the water column.

In this study, we aim to resolve these knowledge gaps in Sumatra, Indonesia, by quantifying the riverine carbon export and investigate the estuarine and marine processes to better understand the fate of terrestrial carbon in the coastal ocean.

## Results and Discussion

### Riverine carbon processes and exports

**Figure 1:**
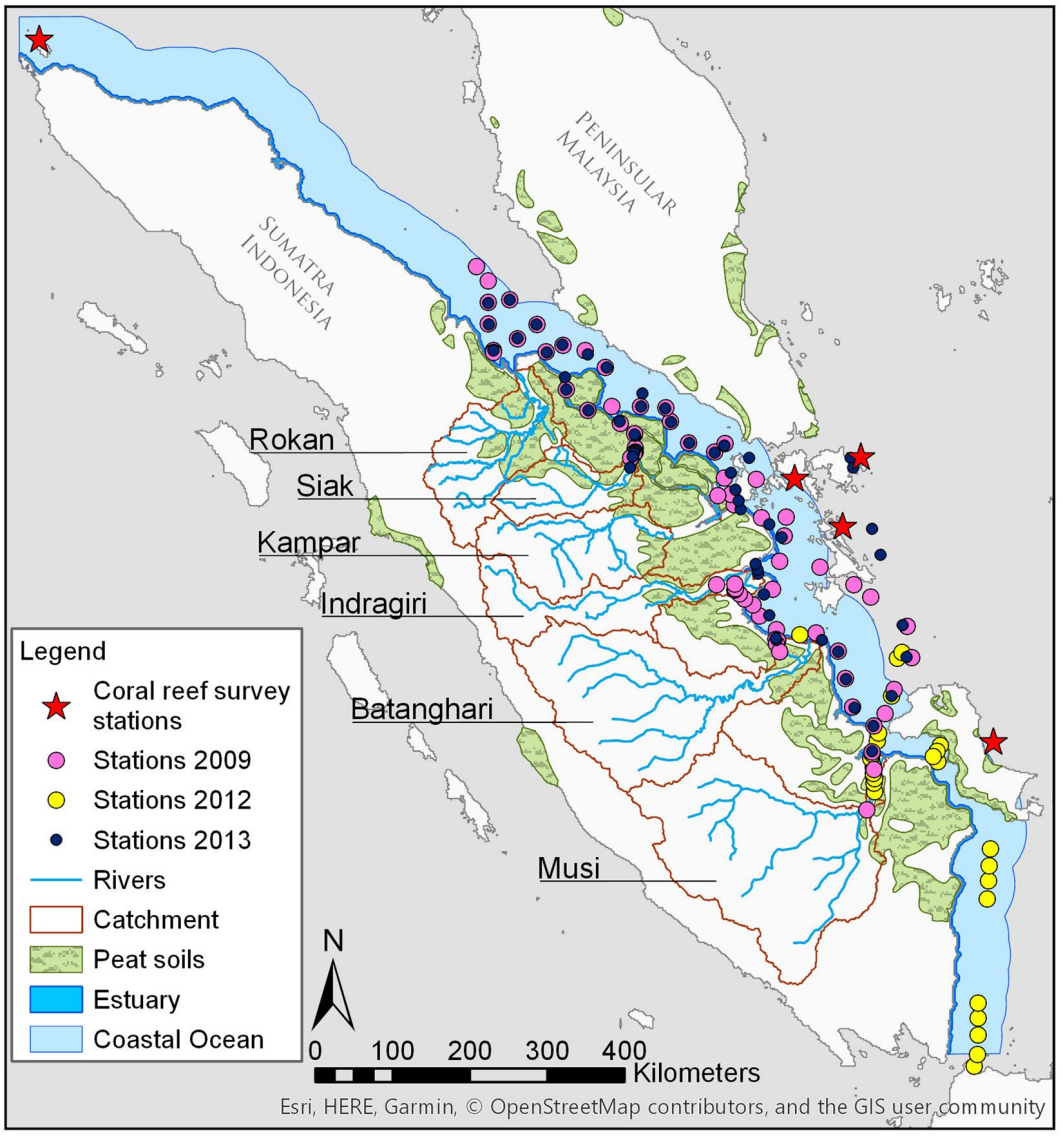
Study area and sample stations in 2009, 2012 and 2013 along the coast of Sumatra, Indonesia. The map was drawn using ArcGIS 10.4.1 by ESRI (http://desktop.arcgis.com/en/).

**Figure 2:**
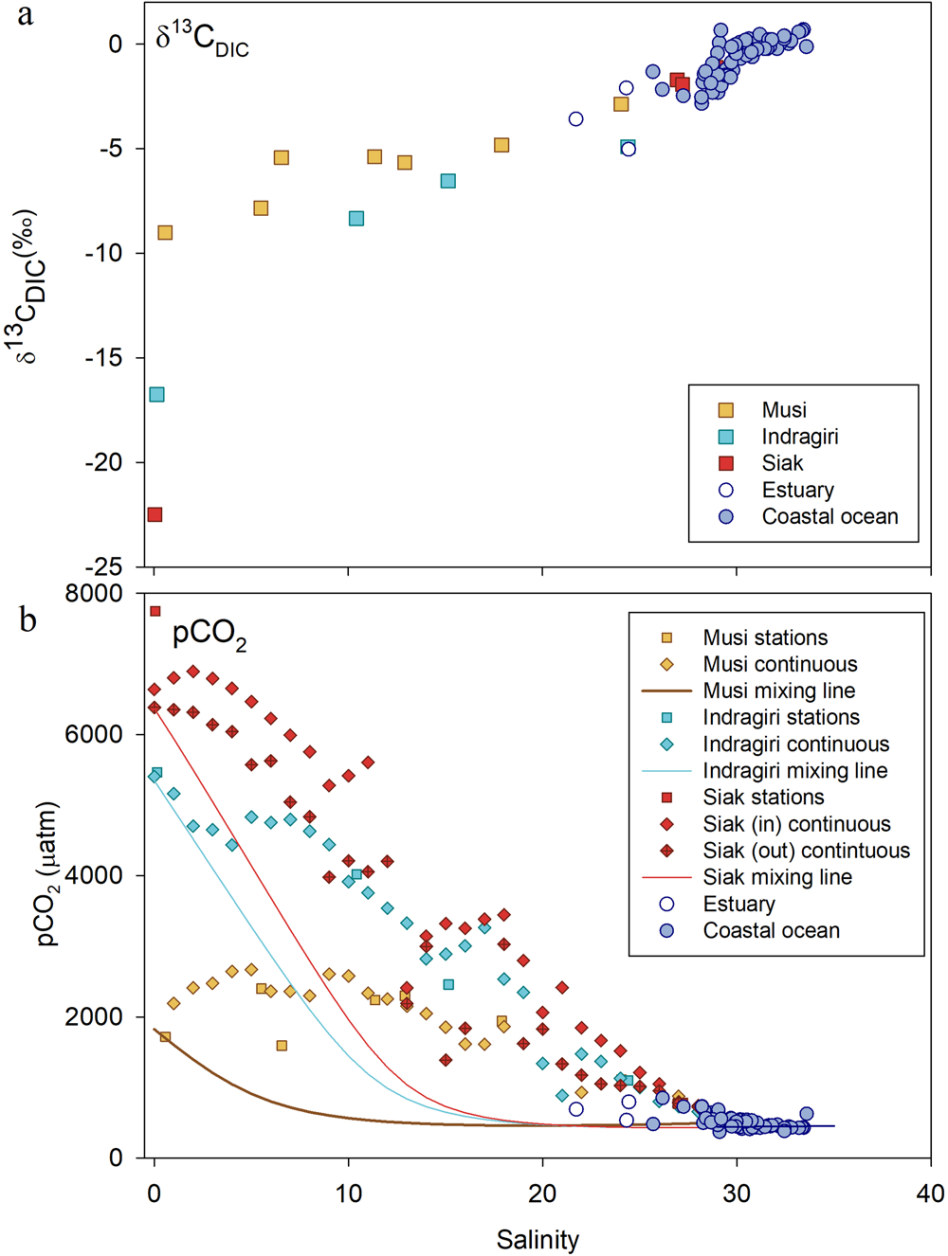
(**a**) δ^13^C_DIC_ values plotted against salinity. (**b**) Measured pCO_2_ concentrations (dots) and expected pCO_2_ concentrations during mixing in the estuaries (mixing lines). To complement the figure with respect to pCO_2_ measurements in the estuaries, the continuous pCO_2_ measurements were used to calculate the average pCO_2_ at each salinity point in the respective rivers (continuous). As the trip in the Siak river covered two days with a pause upstream, the pCO_2_ data is divided between ‘In’ and ‘Out’ to highlight the difference in pCO_2_ concentrations. During the way into the Siak the pCO_2_ concentrations are slightly lower than during the way out. This is presumably due to a plankton bloom that occurred as relatively low DOC concentrations allowed for enhanced light availability, which absorbed CO_2_ for photosynthesis^7^.

Tropical peatlands in Sumatra cover approximately 15.6%^12^ or 72,431 km^2^ of the land area with a thickness between 2 and 10 m^13^ and are mostly located on the coastal plains. The large majority of these peatlands is disturbed as a consequence of deforestation and drainage to make way for agricultural cropland and in particular palm oil plantations^12^, with only a small portion (6%) of pristine tropical peatlands remaining in Southeast Asia^14^. A total of three expeditions were carried out along the north and east coast of Sumatra in October 2009, October 2012 and April 2013, with 72, 32 and 57 sampling stations, respectively. Six rivers were investigated, namely the Musi, Batanghari, Indragiri, Kampar, Siak and Rokan (Fig. 1), of which the river catchments contain various amounts of peatland coverage, ranging from 3.5% in the Musi up to 30.2% in the Rokan catchment (Table 1). Through leaching, carbon is mobilized from the peat soils into the rivers, which is further enhanced through disturbance^6^ and the labile content of the leaves of secondary vegetation^5^. The export ratio between total organic carbon and total inorganic carbon (TOC:TIC) increases with increasing peat coverage as the overlying peat soils leach organic carbon and simultaneously reduce the contribution of dissolved (inorganic) carbonate derived from weathering of underlying mineral soils to the rivers^15,16^. The relative importance of dissolved inorganic carbon (DIC) derived from respiration and silicate weathering can also be quantified by δ^13^C_DIC_. The riverine δ^13^C_DIC_ is a mixture between low δ^13^C_CO2_ values from decomposed plant material, which amounts to an average of −28.0 ± 1.5‰ as derived from leached terrestrial dissolved organic carbon (DOC) measured in the Siak, Rokan and Kampar rivers in 2006, and HCO_3_^−^ derived from weathering of mineral soils^17^, which have an isotopic signature of about 0‰^18^. The Siak river has a δ^13^C_DIC_ of −22.5‰ derived from decomposed terrestrial DOC and less from weathering and its DIC is composed primarily of CO_2_ (Fig. 2a, Table 1 and Supplementary Fig. 1). In the Musi and Indragiri, which have smaller relative peat coverages, δ^13^C_DIC_ values are higher with −16.8‰ and −9.0‰, respectively, which indicates that mineral soil weathering plays a more active role in these catchments.

**Table 1:** Carbon export rates from rivers in Sumatra based on averaged concentrations measured during expeditions from 2009 to 2013, including POC and PIC data from cruises in 2004, 2005, 2006 and 2008. Errors are represented as the standard error.

Location		Musi	Batanghari	Indragiri	Kampar*	Siak	Rokan*	Sumatra*
Catchment	km^2^	56931	44890	17968	26195	10423	19258	464301
River area	km^2^	245	269	174	210	81	154	3714
Discharge	m^3^ s^−1^	3961 ± 587	2309 ± 182	1339 ± 89	2063 ± 297	720 ± 74	1506 ± 307	31820 ± 3606
Peat cover	%	3.5	5	11.9	22.4	21.9	30.2	15.6
Peat cover	km^2^	1993	2245	2138	5868	2283	5816	72431
δ^13^C_DIC_	‰	−9.0	—	−16.8	—	−22.5	—	—
CO_2-DIC_	%	17.1	—	41.9	—	88.3	—	—
HCO_3-DIC_	%	82.7	—	58.0	—	11.7	—	—
CO_3_^2−^_DIC_	%	0.2	—	0.0	—	0.0	—	—
DOC conc.	μM	303 ± 61	311 ± 39	757 ± 99	1280 ± 63	1900 ± 640	781 ± 53	890 ± 159
DOC yield	g C m^−1^ yr^−1^	8.00 ± 3.91	6.05 ± 0.62	21.37 ± 4.99	32.65 ± 0.88	49.71 ± 27.26	23.13 ± 3.09	23.09 ± 6.79
DOC flux	Tg C yr^−1^	0.46 ± 0.22	0.27 ± 0.03	0.38 ± 0.09	0.86 ± 0.02	0.52 ± 0.28	0.45 ± 0.06	10.72 ± 0.12
DIC conc.	μM	748 ± 42	671 ± 21	409 ± 12	294 ± 21	291 ± 11	333 ± 21	483 ± 137
DIC yield	g C m^−1^ yr^−1^	19.71 ± 0.16	13.08 ± 0.03	11.53 ± 0.02	7.49 ± 0.09	7.62 ± 0.03	9.86 ± 0.13	12.53 ± 0.40
DIC flux	Tg C yr^−1^	1.12 ± 0.01	0.59 ± 0.00	0.21 ± 0.00	0.20 ± 0.00	0.08 ± 0.00	0.19 ± 0.00	5.82 ± 0.19
POC conc.	μM	143 ± 51	109 ± -	482 ± 113	156 ± 39	499 ± 109	1034 ± 18	404 ± 55
POC yield	g C m^−1^ yr^−1^	3.76 ± 0.20	2.12 ± -	13.60 ± 0.21	3.98 ± 0.17	13.06 ± 0.29	30.64 ± 0.11	10.48 ± 0.16
POC flux	Tg C yr^−1^	0.21 ± 0.01	0.10 ± 0.00	0.24 ± 0.00	0.10 ± 0.00	0.14 ± 0.00	0.59 ± 0.00	4.87 ± 0.08
PIC conc.	μM	8 ± 0.17	10 ± 0.20	57 ± 1.14	—	0 ± 0.00	—	19 ± 0.38
PIC yield	g C m^−1^ yr^−1^	0.22 ± 0.00	0.20 ± 0.00	1.60 ± 0.00	—	0.00 ± 0.00	—	0.49 ± 0.00
PIC flux	Tg C yr^−1^	0.01 ± 0.00	0.01 ± 0.00	0.03 ± 0.00	—	0.00 ± 0.00	—	0.23 ± 0.00
TOC exp.	Tg C yr^−1^	0.67 ± 0.23	0.37 ± 0.03	0.63 ± 0.09	0.96 ± 0.03	0.65 ± 0.29	1.04 ± 0.06	15.59 ± 0.19
TIC exp.	Tg C yr^−1^	1.13 ± 0.01	0.60 ± 0.00	0.24 ± 0.00	0.20 ± 0.00	0.08 ± 0.00	0.19 ± 0.00	6.04 ± 0.19
TC exp.	Tg C yr^−1^	1.80 ± 0.24	0.96 ± 0.03	0.86 ± 0.09	1.16 ± 0.03	0.73 ± 0.29	1.23 ± 0.06	21.63 ± 0.38
CO_2_:C_exp_ ratio	—	27:73	29:71	43:57	52:48	38:62	49:51	43:57
OC:IC ratio	—	37:63	38:62	73:27	83:17	89:11	85:15	72:28

*DIC concentrations for the Kampar, Rokan and Sumatra were derived from the correlation between DIC concentrations and peat coverage of the Musi, Batanghari and Siak rivers.

The dissolved and particulate organic and inorganic riverine carbon fluxes have been calculated based on measurements carried out between 2004 and 2013 (Supplementary Table 1) and were interpolated to encompass Sumatra. Exports were calculated by multiplying the average carbon end-member concentration with discharge, which was based on averaged monthly precipitation rates (Supplementary Table 1) and assuming an evapotranspiration rate of 37.9%^6^. DOC and DIC end-member concentrations per expedition were derived through correlating their concentrations with salinity, where the y-intercept at salinity 0 represents the end-member concentration. DIC concentrations (μmol kg^−1^) were calculated using the co2sys program with total alkalinity (TA) and pCO_2_ measurements as input parameters, alongside other required inputs of salinity, temperature, pressure and nutrient measurements. Particulate organic carbon (POC) and particulate inorganic carbon (PIC) concentrations were derived from stations in a salinity range of 0–1, as it was not possible to obtain an end-member due to few data points. Exports normalized to catchment area represent the yield.

On average 72% of the exported carbon from Sumatra is organic with 10.72 ± 0.12 Tg C yr^−1^ of DOC and 4.87 ± 0.08 Tg C yr^−1^ of particulate organic carbon (POC), adding up to 15.59 ± 0.20 Tg C yr^−1^ of TOC (Table 1). The export of TIC amounts to 6.05 ± 0.19 Tg C yr^−1^, mostly consisting of DIC with 5.82 ± 0.19 Tg C yr^−1^, whereas particulate inorganic carbon (PIC) contributes on a marginal level with 0.23 ± 0.00 Tg C yr^−1^. As a whole, Indonesia, with a peat coverage of 11.9% or 2.3*10^5^ km^2^ (ref.^7^), has a TOC export of 53.06 Tg C yr^−1^ and a TIC export of 25.65 Tg C yr^−1^. In comparison, the Amazon river has riverine carbon exports of 36 Tg C yr^−1^ of TOC and 35 Tg C yr^−1^ of TIC^19^, which shows that Indonesia, regardless of its relatively small size of 1.9 million km^2^ (ref.^7^) compared to the Amazon catchment of 3.9 million km^2^ (ref.^19^), plays a crucial role with respect to carbon exports. Its relatively large riverine export of organic carbon shifts the attention to the fate of this organic carbon in the estuaries.

### Carbon fluxes and processes in the estuaries and coastal ocean

As river waters reach the estuaries and coastal ocean of Sumatra, pCO_2_ levels rapidly decrease. In order to investigate to which extent mixing with low pCO_2_ ocean waters is responsible for this measured decrease, pCO_2_ mixing lines were calculated. In the estuaries, TA and DIC concentrations increase in a linear fashion as low concentration river waters mix with high concentration coastal ocean waters. This linear relationship along a salinity gradient is referred to as a mixing line and represents the expected TA and DIC concentrations at a specific salinity as a consequence of mixing (Supplementary Fig. 2). Based on these TA and DIC mixing lines calculated for the Musi, Indragiri and Siak estuaries, pCO_2_ mixing lines were calculated using co2sys with the TA and DIC concentrations of the mixing lines as input parameters, in addition to temperature, salinity and air pressure. The pCO_2_ (non-linear) mixing lines represent the expected decrease in pCO_2_ concentrations along the salinity gradient caused by mixing and are visualized along with the measured pCO_2_ concentrations for the Musi, Indragiri and Siak estuaries in Fig. 2b. The pCO_2_ mixing lines are initially relatively high in accordance with the measurements, but quickly decrease exponentially. However, the higher pCO_2_ measurements indicate that additional CO_2_ is produced that increases the pCO_2_ beyond the level expected during mixing. This confirms the general observation that especially the estuaries are heterotrophic systems, where respiration and decomposition of organic carbon are dominant processes. The δ^13^C_DIC_ data (Fig. 2a) shows the influence of terrestrial carbon in the estuaries and coastal ocean. However, its increase with increasing salinity is in a linear fashion, thereby seemingly due to mixing. Decomposition of terrestrial organic carbon would result in more negative values, expressed in a curve below the observed linear correlation. On the other hand, outgassing of CO_2_ at a pCO_2_ twice or more that of the atmosphere raises the δ^13^C_DIC_ values^20^, providing a curve above the observed correlation. Both processes occur in the estuaries and balance the resulting δ^13^C_DIC_ values to appear linear.

In order to quantify how much of the respired exported carbon is emitted as CO_2_ to the atmosphere, the CO_2_ yields of the estuaries and coastal ocean were calculated and amount to 670.7 ± 98.4 g C m^−2^ yr^−1^ and 49.4 ± 7.2 g C m^−2^ yr^−1^, respectively (Table 2). Multiplied by their surface areas, this results in a CO_2_ flux of 7.3 ± 1.1 Tg C yr^−1^ in the estuaries and 6.3 ± 0.9 Tg C yr^−1^ in the coastal ocean. This finding is contradictory to a recent study that predicts the coastal ocean of Southeast Asia to be a carbon sink^21^. Whereas this may be the case for the northern part of Southeast Asia from where this data point is extrapolated due to data scarcity, our data shows that the coastal ocean of Sumatra is instead a carbon source. Indeed, it appears that the short residence time of the rivers creates relatively modest CO_2_ emissions in the rivers with 16.5 Tg C yr^−1^ in Sumatra^7^, but in fact shifts this process of CO_2_ emission to the estuaries and coastal ocean where the exported organic carbon is respired, thereby causing a combined CO_2_ emission of 13.6 ± 2.0 Tg C yr^−1^. In regard to the total carbon export of 21.6 Tg C yr^−1^, this suggests that the excess of 8.0 Tg C yr^−1^ is either exported into the sediments or it is adsorbed by marine waters. Although actual sedimentation rates are uncertain, globally it is estimated that approximately 10% of the exported terrestrial organic carbon is sequestered in continental margin sediments^22^. Assuming a similar burial rate for Sumatra and its TOC export of 15.6 Tg C yr^−1^, this results in a sedimentation rate of 1.6 Tg C yr^−1^, which would reduce the excess of exported carbon from 8.0 to 6.4 Tg C yr^−1^. The remaining excess of carbon may be partially exported to the open ocean^23^, but is most presumably further remineralized to form CO_2_ (ref.^24^). In addition to the exported DIC_CO2_, this may be used for photosynthesis or alternatively is absorbed by marine waters through carbonate dissolution, which shifts the carbonate system towards lower CO_2_ concentrations.

**Table 2:** CO_2_ outgassing fluxes of the rivers, estuaries and coastal ocean of Sumatra based on averaged concentrations measured during expeditions from 2009 to 2013. Errors are represented as the standard error.

Location		Estuaries	Coastal ocean	Subtotal marine
Area	km^2^	10818	127674	138492
Wind speed	m/s	5.59 ± 0.41	5.59 ± 0.41	/
K_W_	cm hr^−1^	12.0 ± 1.8	12.0 ± 1.8	/
pCO_2_ conc.	μatm	2038 ± 56	554 ± 1	/
CO_2_ yield	g C m^−2^ yr^−1^	670.7 ± 98.4	49.4 ± 7.2	/
CO_2_ flux	Tg yr^−1^	7.3 ± 1.1	6.3 ± 0.9	13.6 ± 2.0

K is based on Wanninkhof. The spread of the K_W_, CO_2_ yields and fluxes are best/worst case scenarios, calculated based on the s.d. of the wind speed. The spread of the pCO_2_ is the s.e.

### Carbonate dissolution

**Figure 3:**
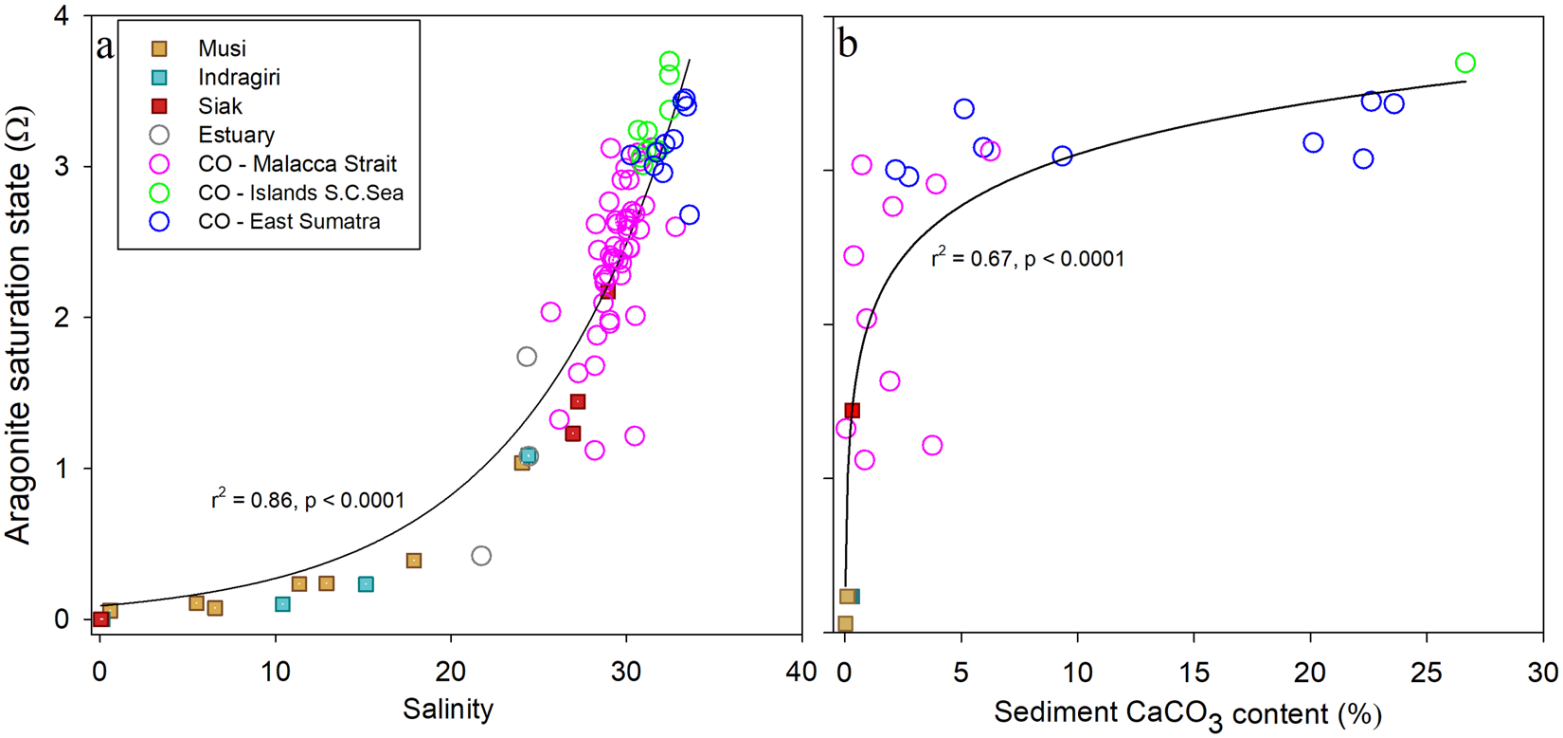
(**a**) Aragonite saturation states against salinity for the estuaries, coastal ocean and three regions of the coastal ocean of Sumatra: Malacca Strait, Tudjuh Islands and East Sumatra. (**b**) Correlation between the aragonite saturation states and sediment CaCO_3_ content.

**Figure 4:**
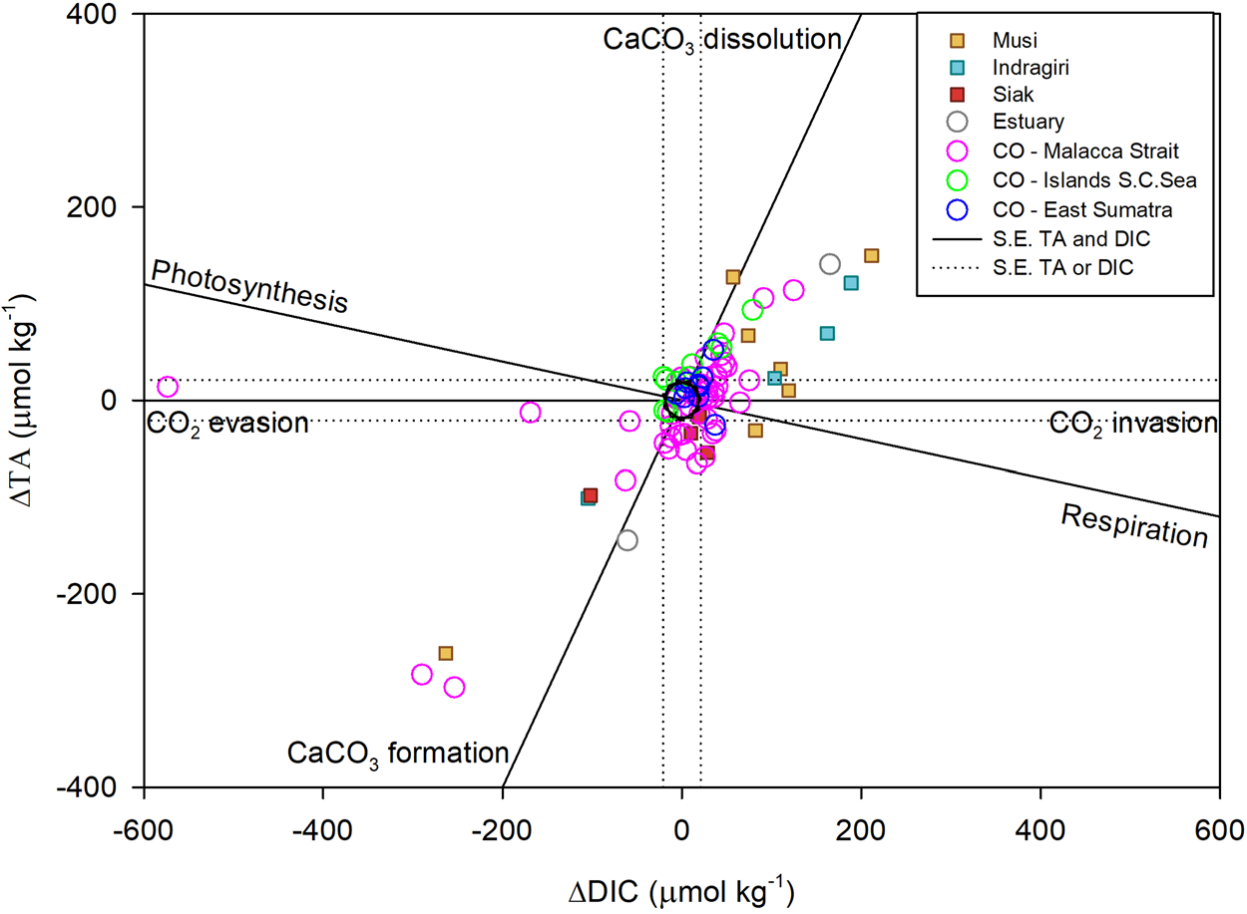
Deviations of DIC and TA for the Musi, Indragiri and Siak estuaries, as well as other estuaries and the coastal ocean. The black solid lines show the processes that occur corresponding to the ratio of increase or decrease of DIC and TA, namely photosynthesis/respiration, CO_2_ invasion/evasion and CaCO_3_ formation/dissolution. Data points within the black standard error (s.e.) circle have no significant deviations for both TA or DIC and indicate the process of mixing, whereas the direction and degree of deviation away from the s.e. circle point to other processes as indicated by the black solid lines in the plot. Data points in between lines show a mixture of the neighboring processes. Modified after Zeebe & Wolf-Gladrow (2001).

Marine organisms primarily use two major forms of CaCO_3_, namely aragonite (corals and many mollusks) and calcite (coccolithophores, foraminifera and some mollusks)^25^. The saturation states of aragonite (Ω_AR_) and calcite (Ω_CA_), calculated using co2sys with TA and pCO_2_ as input parameters, found in the Sumatran estuaries range between 0–1.1, which encourages carbonate dissolution^25^ and hampers the growth of carbonate-producing organisms. In the coastal ocean, the values increase up to 3.5 for Ω_AR_ and 5.5 for Ω_CA_ (Fig. 3a and Supplementary Fig. 2, respectively). This gradient from the estuaries to the coastal ocean is notably reflected by the mollusk species richness and abundance, which is correlated to the sediment carbonate weight content^26^. Indeed, the minor sediment carbonate content in the river mouths (0–4%) and Malacca Strait (<1%) allow few mollusk species to thrive with very low abundance, whereas the increased carbonate content in East Sumatra (2–79%) and the Tuju Islands (27–92%) show increased species richness and mollusk abundance^26^. The sediment carbonate content is also correlated to the Ω, where the increase of carbonate content is translated in a relatively quick increase in Ω_AR_ and stabilizes between 3 and 4 as the carbonate content reaches beyond 25% (Fig. 3b). Nonetheless, taking into account that calcification rates and cementation sharply decrease at Ω_AR_ < 4 (ref.^27^) and Ω_AR_ < 3 (ref.^28^), respectively, the findings indicate that calcification rates in the coastal ocean are not optimal. In order to visualize the balance between CaCO_3_ dissolution and formation, the measured total alkalinity (TA) and DIC concentrations were compared to their respective mixing lines (Supplementary Figs 3 and 4). The DIC and TA deviations were then plotted against each other to visualize their ratio, which sheds light on the occurring processes (Fig. 4). In accordance with their low Ω_AR_, most of the estuarine data points show the largest dissolution and are offset to the right due to additional DIC in the form of respired CO_2_. This confirms our previous observation that, besides CaCO_3_ dissolution as evident from this plot and the Ω_AR_/Ω_CA_, respiration is an active process in the estuaries. Whereas optimal Ω_AR_ and Ω_CA_ conditions in the coastal ocean would result in its data points along the CaCO_3_ formation, the cluster is shifted toward dissolution instead, with an offset towards respiration. This indicates that ocean acidification as a consequence of oversaturation of respired CO_2_ is taking its toll in the coastal ocean as well, through reduced calcification rates, but also carbonate dissolution. Although the carbonate source for dissolution in the estuaries consists of sediment and benthic calcifying organisms, such as mollusks, it may also affect coral reefs located a bit further out the coast as the situation persists or aggravates. Indeed, whereas a simultaneous study revealed global reefs situated in the open ocean to show net carbonate formation^29^, a patch at the northwestern tip of Sumatra was recently identified as ‘dark spot’, distinguished by intensive fishing technologies, but also environmental shocks such as coral bleaching^30^. In addition, a recent survey in the coastal ocean of Sumatra has revealed that the status of coral reefs at 81% of 75 clustered survey stations (Fig. 1) is fair to bad, meaning that less than 50% of the corals is alive^31^, although the precise cause for this status is uncertain. However, knowing that ocean acidification, which lowers the saturation states, may cause coral bleaching and productivity loss^32^, and seeing that the surveys were at the outskirts of the study area, coral reefs and other calcifying organisms closer to the coast may be in even worse condition and are at a higher risk to bleaching, reduced calcification rates and dissolution caused by lowered saturation states as a consequence of enhanced riverine carbon inputs, as already apparent in the mollusk assemblages^26^. The disappearance of carbonate-producing reef organisms would have vast ecological and economic impacts as the trophic system becomes disturbed, thereby affecting biodiversity, sediment production and sequestration, in addition to destabilization of the coast^33^.

### The invisible carbon footprint

**Figure 5:**
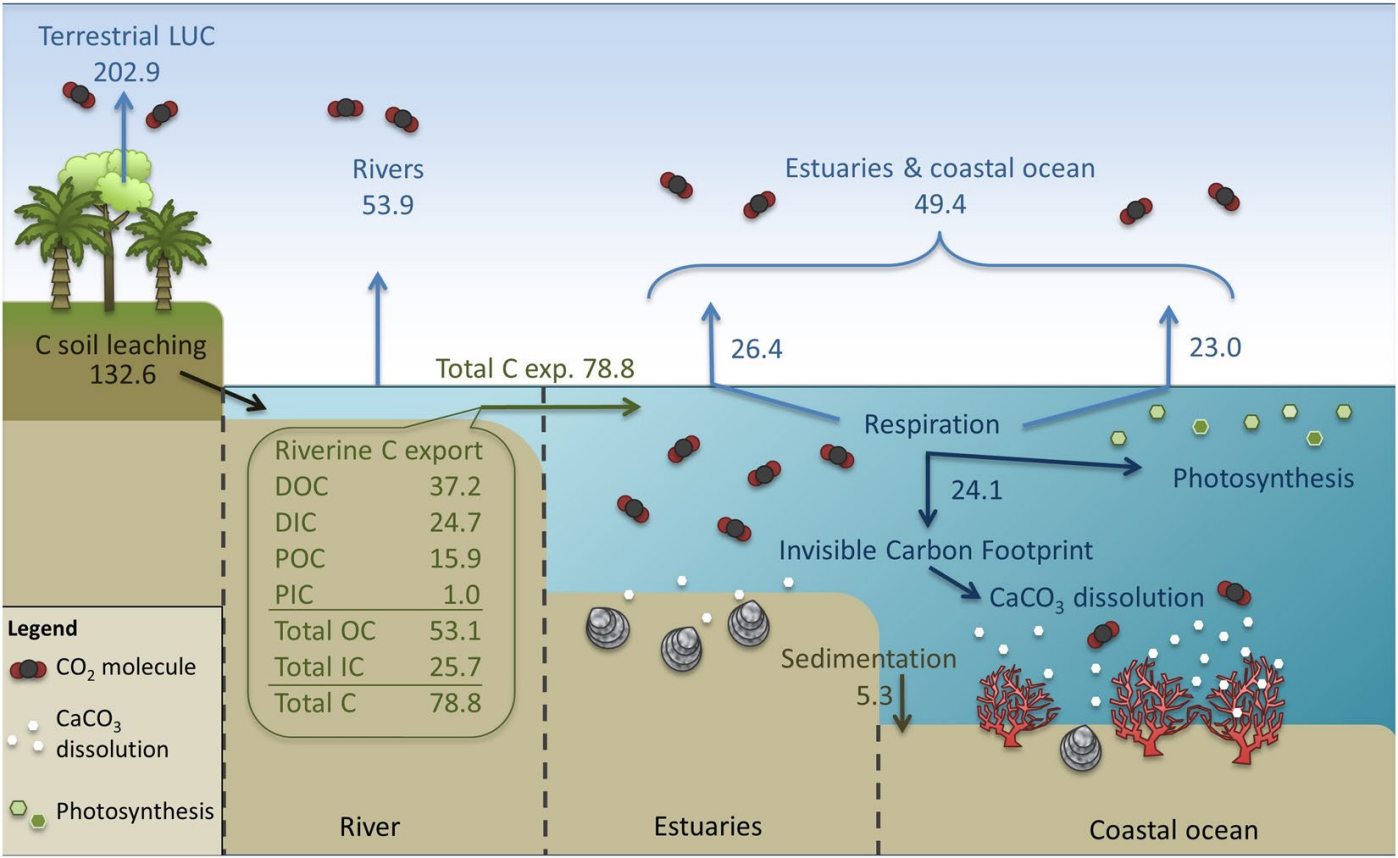
Overview of the carbon fluxes (Tg C yr^−1^) in the rivers, estuaries and coastal ocean in Indonesia. With the exception of the terrestrial LUC emissions, all fluxes include a portion of the natural indirect background emission from pristine peatlands of 14.4 Tg C yr^−1^ and the indirect background emission from non-peatlands of 13.9 Tg C yr^−1^, which amounts to 28.3 Tg C yr^−1^ in total.

In Sumatra 62.7% of the exported total carbon (13.6 of 21.6 Tg yr^−1^) is emitted from the estuaries and coastal ocean and 10% of its organic carbon export is assumed to be sequestered in the sediments. Adopting similar emission and burial percentages for entire Indonesia with its total carbon export of 78.8 Tg C yr^−1^ and organic carbon export of 53.1 Tg C yr^−1^, the CO_2_ emissions from the estuaries and coastal ocean result in a total of 49.4 Tg C yr^−1^ with another 5.3 Tg C yr^−1^ buried in the sediments (Fig. 5). The remaining exported carbon of 24.1 Tg C yr^−1^ (30.6%) remains in the marine waters where it further respires and favors CaCO_3_ dissolution by lowering the saturation states. As saturation states are lowered by inputs of Ca^2+^-poor and acidic freshwater, this situation is induced by the peatlands, which overlie mineral soils thereby reducing weathering, and moreover produce an acidic CO_2_-rich environment through decomposition of leached organic carbon in the rivers that extends into the estuaries and beyond. Having greatly increased DOC leaching from peat soils through degradation by 200% as compared to the natural situation^5^, anthropogenic disturbance may therefore be seen as a primary contributor to the low saturation states in the estuaries and, to a lesser extent, the coastal ocean. The respired carbon that remains in the ocean and favors carbonate dissolution can therefore be viewed as the invisible carbon footprint induced by LUC, which should be considered in terms of ocean acidification. UNESCO already recognizes ocean acidification as a serious threat and urges the UNFCCC to consider its negative effects on the ocean chemistry and marine ecosystem^34^. In addition, the UN Sustainable Development Goal 14 ‘Life below water’^35^ aims to sustainably use marine resources and address the impact of ocean acidification. However, whereas this study shows that the effects of LUC stretch beyond the terrestrial realm, the invisible carbon footprint is currently overlooked in mitigation policies alongside the CO_2_ emissions from the rivers, estuaries and coastal ocean, as the focus is on reducing direct terrestrial carbon emissions^36–38^.

In Southeast Asia, approximately 6% of the peatlands remain pristine, whereas 11% is covered by disturbed (secondary) vegetation, 2% is (seasonal) water surface and 81% is degraded and converted peatland cover^14^. Assuming an even distribution, a peatland cover of 2.3 *10^5^ km^2^ and a yield of 433 g C m^−2^ yr^−1^ (ref.^4^), the direct terrestrial CO_2_ emission from secondary vegetation in Indonesia amounts to 10.9 Tg C yr^−1^. Its direct terrestrial CO_2_ emissions due to LUC via peat oxidation and fires from the degraded and converted peatland cover result in 109.9 Tg yr^−1^ (ref.^39^) and 82.1 Tg C yr^−1^ (ref.^40^), respectively, which amounts to a total direct terrestrial CO_2_ emission of 202.9 Tg C yr^−1^. Carbon loss due to indirect aquatic emissions from the rivers 53.9 Tg yr^−1^, (ref.^7^), estuaries and coastal ocean (49.4 Tg yr^−1^), as well as the invisible carbon footprint (24.1 Tg C yr^−1^), amounts to 127.4 Tg C yr^−1^.

However, this value still includes the natural background indirect emissions from pristine peatland and non-peatlands. The natural indirect peatland emission can be deduced from the TOC leaching rate of pristine peatlands of 63 g C m^−2^ yr^−1^ (ref.^6^) and amounts to 14.4 Tg C yr^−1^. The indirect emission from non-peatland (1.7 *10^6^ km^2^) is deduced from the correlation with peat coverage and DOC, where a peat coverage of 0% results in a DOC leaching of 5.6 g C m^−2^ yr^−1^ or 9.5 Tg C yr^−1^. POC values vary per river in a non-correlating fashion, but POC exports consist on average of 45% of the DOC export, resulting in an indirect POC leaching rate of 2.6 g C m^−2^ yr^−1^ or 4.4 Tg C yr^−1^ with a TOC emission from non-peatland of 13.9 Tg C yr^−1^.

The total natural indirect TOC emission from peat (14.4 Tg C yr^−1^) and non-peatlands (13.9 Tg C yr^−1^) amounts to 28.3 Tg C yr^−1^. Subtracting the natural indirect emission of 28.3 Tg C yr^−1^ from the current one of 127.4 Tg C yr^−1^ results in a total indirect LUC emission of 99.1 Tg C yr^−1^. This indicates that LUC has not only increased soil DOC leaching by 200%^5^ but also POC leaching, which leads to increased soil TOC leaching or indirect emissions by 350% as compared to the natural situation.

Including the direct terrestrial peatland emissions of 202.9 Tg C yr^−1^, the total carbon loss due to LUC amounts to 302.0 Tg C yr^−1^, which represents an increase of 49%.

Considering this large impact along with the environmental and economic effects on global climate and the marine ecosystem, it is of vital importance that LUC mitigation policies are not only limited to direct terrestrial greenhouse gas emissions, but also incorporate the aquatic and marine CO_2_ emissions as well as the invisible carbon footprint.

## Methods

### Climate study area

Sumatra is subject to the Malaysian-Australian monsoon as a consequence of the meridional variation of the intertropical convergence zone. During the wet season, which lasts from October to April, the monsoon brings heavy rains from the north, whereas from May to September dry air currents from Australia induce a dry season^41^. Precipitation rates vary between 123 mm in July to 312 mm in November with an annual sum of 2,696 mm in Pekanbaru, Central Sumatra^42^.

### Sampling methods

Salinity, pCO_2_ and temperature were measured continuously by means of underway instruments, which were connected via a through-flow system and supplied with surface water from an approximate depth of 1 m. Salinity was measured using a Seabird SBE 45 Micro TSG sensor, whereas temperature was measured via the integrated sensor of the Meinsberg EGA 140 SMEK pH sensor. pCO_2_ measurements were carried out with a Li-Cor 7,000 pCO_2_ analyzer in 2009 and 2012, and a Contros HydroC CO_2_ Flow Through Sensor in 2012 and 2013. Both pCO_2_ devices were calibrated prior to the expeditions, of which the Contros HydroC device at 100, 448 and 800 ppm. The Li-Cor 7,000 device was calibrated using certificated NOAA reference gases (#CB08923 with 359.83 ppm, #CA06265 with 1,021.94 ppm) and another certificated calibration gas with 8,000 ppm. Wind parameters were measured using pre-installed equipment available on the vessel in 2009 and by means of a Lambrecht Ultrasonik anemometer in 2012, both at a height of 10 m above sea level.

In addition to continuous measurements, water samples were taken at each station using a Niskin bottle at circa 1.5 m depth. After a total storage (during and after expedition) of maximum three weeks, the samples were analyzed in the laboratory in Bremen, Germany. Samples for δ^13^C_DIC_ were stored in amber-colored 20 ml bottles, deprived of air, intoxicated with mercuric chloride (HgCl_2_) and analysed using the Finnigan GasBench II. In this instrument, organic compounds eluting from a GC column are converted into simple gases when traversing a capillary micro-reactor. Accordingly, all compound specific isotope ratios are analyzed in the IRMS. DOC samples were filtered (0.45 μm) into 60 ml high-density polyethylene (HDPC) bottles and acidified with phosphoric acid (20%) up to pH 2.0. By means of a Shimadzu TOC-VCPH Total Organic Carbon Analyzer, the samples were combusted at 680 °C within a quartz column and the released CO_2_ was measured using the oxidative combustion-infrared analysis. The relative standard error for the method was ±1%. Alkalinity samples were collected in 250 ml glass bottles in 2009 and 2012 and in 125 ml LDPE flasks in 2013, deprived of air, intoxicated with HgCl_2_ and analyzed using a VINDTA 3 S instrument. Known amounts of sampled seawater were titrated with constant increments of 0.15 ml of hydrochloric acid (HCl) until a total amount of 4.2 ml HCl was reached. The HCl in the device was calibrated with a sodium chloride solution to approximate the ionic strength of seawater. The process of the open cell titration allowed the assumption that the total amount of DIC was approximately zero in the pH region of 3.0–3.5. The process was monitored using a pH glass electrode cell and the total alkalinity (TA) was calculated from the titrant volume and electromotoric force using a non-linear least-squares approach that corrected for the reactions with sulphate and fluoride ions. Measurements of temperature, salinity, pH, pCO_2_ and TA at the stations are shown in Supplementary Table 3.

### CO_2_ flux and piston velocity calculations for estuaries and coastal ocean

CO_2_ fluxes (F) were calculated from the pCO_2_ measurements of the continuous data using:

1 $${\rm{F}}={{\rm{K}}}_{{\rm{CO}}2}\,\times \,{{\rm{K}}}_{0}\,\times \,{{\rm{\Delta }}\mathrm{pCO}}_{2}$$

where K_CO2_ is the CO_2_ piston velocity, K_0_ the solubility of CO_2_ in seawater^43^ and ΔpCO_2_ is the sea-air pCO_2_ difference with an average atmospheric CO_2_ concentration of circa 390 ppm, as measured during the cruises.

Although piston velocities are affected by many processes such as surface wave types, formation of air bubbles, humidity and temperature gradients and organic film coating, in the coastal systems and ocean piston velocities are primarily influenced by wind speed and the Schmidt number^44^. Therefore, piston velocity calculations related to wind speed have been chosen for this study. As calculations from Wanninkhof^45^ and Nightingale *et al*.^46^ are widely used in the literature^47,48^, both formulas have been used to calculate K_CO2_ for comparative matters. CO_2_ calculations based on Wanninkhof’s principles were 9.2% higher than Nightingale’s and are used to represent a maximum estimate, which results in a lower invisible carbon footprint. The results based on Nightingale’s principle are summarized in Supplementary Table 2.

2 $${{\rm{K}}}_{{\rm{W}}92}=0.31\,\ast \,{{\rm{U}}}^{2}\,\ast \,{(\mathrm{Sc}/600)}^{-0.5}$$

3 $${{\rm{K}}}_{{\rm{N}}}=(0.222\,\ast \,{{\rm{U}}}^{2}+0.333\,\ast \,{\rm{U}})\,\ast \,{(\mathrm{Sc}/600)}^{-0.5}$$

where K_W92_ and K_N_ are the formulas for Wanninkhof and Nightingale, respectively. U is wind speed in m s^−1^ at a height of 10 m above sea level and Sc is the Schmidt number for CO_2_ (kinematic viscosity of water divided by the diffusion coefficient of CO_2_ in water) in seawater determined for temperatures between 0 and 30 °C^45^ calculated by:

4 $${\rm{Sc}}=2073.1+-\,125.62\,\ast \,{\rm{T}}+3.6276\,\ast \,{{\rm{T}}}^{2}+-\,0.043219\,\ast \,{{\rm{T}}}^{3}$$

where T is temperature in °C. Although wind speed was measured during the cruises in 2009 and 2012 with averages of 2.39 ± 0.01 m s^−1^ and 3.97 ± 0.02 m s^−1^, respectively. However, the annual wind speed derived from QuikSCAT^49^ by averaging monthly measurements between 2001 and 2008 within the coastal ocean area (Fig. 1) resulted in an annual average wind speed of 5.59 ± 0.41 m s^−1^. Therefore, the QuickSCAT average was used to get a maximum estimate on emissions.

### Surface area calculations

Catchment areas were defined by means of a relief model in ArcGIS 9.3 with the ArcHydro extension, which was derived from SRTM90m digital elevation model of the Consortium for Spatial Information of the Consultative Group for International Agricultural Research (CGIAR-CSI). Peat coverage in each catchment was determined by overlaying the determined catchment areas by the FAO soil map of the world^16^. The peat coverage for Sumatra was derived from Miettinen *et al*.^12^.

Generally, the border between estuaries and coastal ocean is predetermined at a salinity equal to or more than 30^50^. However, in this study the correlation between salinity and the aragonite saturation state off the coast of Sumatra shows a clear distinction of this border, indicated by Ω_AR_ values of ≤1 below salinity 25, and a rapid increase of Ω_AR_ ≥ 1 at salinities ≥ 25 (Fig. 3a). Therefore, the border between estuaries and coastal ocean is here defined at a salinity equal to or higher than 25. By correlating the salinity and distance to shore, this border is found at an approximate distance of 3 km (Supplementary Fig. 5). Based on this distance, the surface area of the estuaries was estimated using ArcGIS 10.4 and, assuming that estuaries influence the entire coastline, amounts to 10,818 km^2^. The perimeter of the coastal ocean is based on the correlation between salinity and δ^13^C, where the terrestrial influence, characterized as δ^13^C values below the marine δ^13^C signature of circa +1‰ (ref.^51^), reached up to a salinity of circa 32.8 (Fig. 2a). This salinity coincided with a distance of circa 67 km, which resulted in a surface area of 127,674 km^2^.

### Calibration experiment

As pCO_2_ was measured with different devices in 2009 and 2013, a CO_2_ calibration experiment was conducted to validate the Contros measurements, during which different concentrations of CO_2_ gas were delivered using a gas mixing system. The gas concentrations delivered by the gas mixing system were first monitored and compared by the mixing system regulator, the Li-Cor 7000, the Li-820 and the cavity ring-down spectrometer (Picarro G2201-i) in a range from circa 500 to 6000 ppm (Supplementary Fig. 6a). The gas was then used to calibrate seawater in a range of 500–5000 ppm that was pumped into the Li-Cor 7000 equilibrator and the Contros sensor. The measured pCO_2_ concentrations were highly correlated (Supplementary Fig. 6b), especially in the lower concentration range common in the coast, which justified the Contros measurements.

### Uncertainty estimates

The errors associated with the averaged parameters in the rivers, estuaries and coastal ocean are presented as the standard error (s.e.). The error range of the alkalinity is based on the standard error of the seawater standards measured during the sample analysis and applied to the respective samples. The error of DIC can be seen as best/worst case scenario, as the errors of TA and pCO_2_ have been integrated throughout the co2sys calculations. The error range of the CO_2_ yields and fluxes in the estuaries and coastal ocean are the result of the standard deviation of the piston velocities in turn as a consequence of the standard deviation of the wind speed (5.59 ± 0.41 m s^−1^), which was integrated in the CO_2_ yield and flux calculations to give a best/worst case scenario.

## Electronic supplementary material

Supplementary Information

## Data Availability

The datasets generated during and/or analyzed during the current study are available from the corresponding author on reasonable request.
