# Supplementary material for: The Invisible Carbon Footprint as a hidden impact of peatland degradation inducing marine carbonate dissolution in Sumatra, Indonesia
Source: Sci Rep. 2018 Nov 27;8:17403. doi: 10.1038/s41598-018-35769-7 (PMC6258705; doi:10.1038/s41598-018-35769-7)
Supplement: Supplementary file 1 — Supplementary Information [file 41598_2018_35769_MOESM1_ESM.pdf]

Supplementary information

supporting the manuscript:

The Invisible Carbon Footprint as a hidden impact of peatland degradation inducing marine carbonate dissolution in Sumatra, Indonesia

Francisca Wit[1*], Tim Rixen[1,2], Antje Baum[1], Widodo S. Pranowo[3] and Andreas A. Hutahaean[4]

1) *Francisca Wit (*Corresponding author), Leibniz Center for Tropical Marine Research (ZMT), Fahrenheitstrasse 6, 28359 Bremen, Germany (*francisca.wit@leibniz-zmt.de or fmc.wit@gmail.com*)*
2) *Institute of Geology, University of Hamburg, Bundesstrasse 55, 20146 Hamburg, Germany*
3) *Research & Development Center for Marine & Coastal Resources (P3SDLP), Gedung II BALITBANGKP, Jalan Pasir Putih II, Ancol Timur, Jakarta, 14430, Indonesia*
4) *Coordinating Ministry of Maritime Affairs, Jalan. MH. Thamrin No. 8, Jakarta 10340, Indonesia*

Figures and Tables

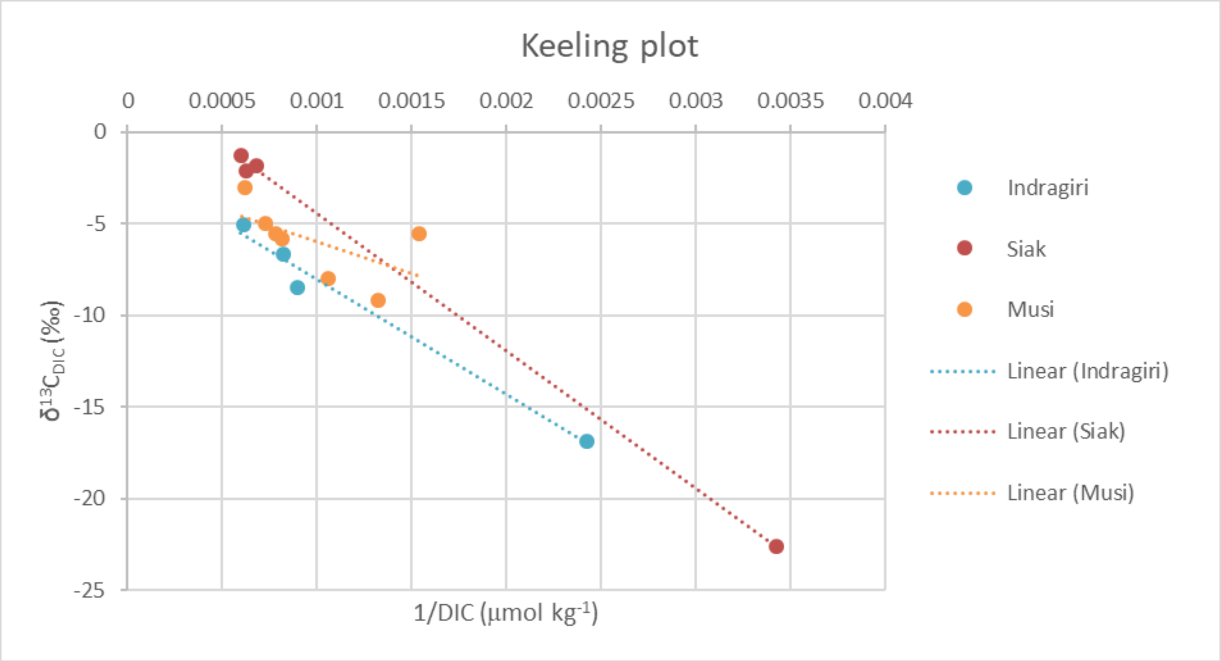

Supplementary Figure 1: Keeling plot showing $\delta^{13}C_{DIC}$ versus 1/DIC concentration. Due to the mixing between river and ocean waters the $\delta^{13}C_{DIC}$ and the 1/DIC data show linear correlation. However, the regression equations obtained from the three studied rivers differ because the end-members vary as well.

Towards the ocean side the Keeling plot shows that the $\delta^{13}C_{DIC}$ in the ocean waters vary. This could e.g. be caused phytoplankton blooms which preferentially take the ^{12}C and thus increases $\delta^{13}C_{DIC}$ in the remaining sea water. Since the Siak is a nutrient-poor black water river the effects are lower than e.g. off the Musi river which is a comparably nutrient-rich normal river characterized by a relatively low peat coverage in its catchment.

The difference between a peat and an almost non-peat-draining river affect also the riverine end-member: The Siak is a black water river characterized by high DOC concentrations and compared to the other rivers low DIC concentrations. Due to the resulting low pH the vast majority of the DIC occurs as CO_2. Since it is produced during the respiration of DOC it shows almost the same $\delta^{13}C$ as peat and plants. The Musi is the other extreme. Accordingly, the DOC concentrations are lower and the dissolution of carbonates rocks in the mineral soils raise the pH and lowers $\delta^{13}C_{DIC}$ because carbonate rocks are of marine origin showing $\delta^{13}C_{DIC}$ values similar to those of ocean waters. Furthermore, due to the higher pH shifts the carbonate system from CO_2 to HCO_3^- which lowers the CO_2 emission and increases in addition to the dissolution of carbonate of the DIC concentration in the river. The $\delta^{13}C_{DIC}$ is this product of mixing between the light respired organic matter and the heavier dissolved carbonate.

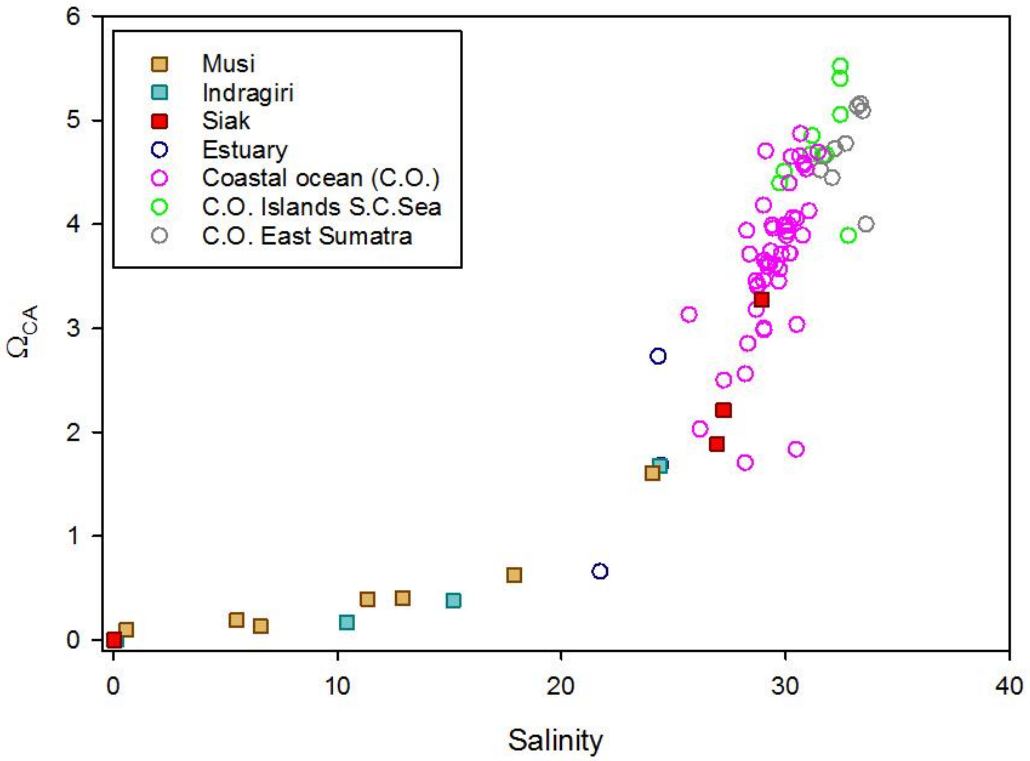

Supplementary Figure 2: Calcite saturation states versus salinity for the estuaries, coastal ocean and three regions of the coastal ocean (C.O.) of Sumatra: the Malacca Strait, islands in the South China Sea and East Sumatra.

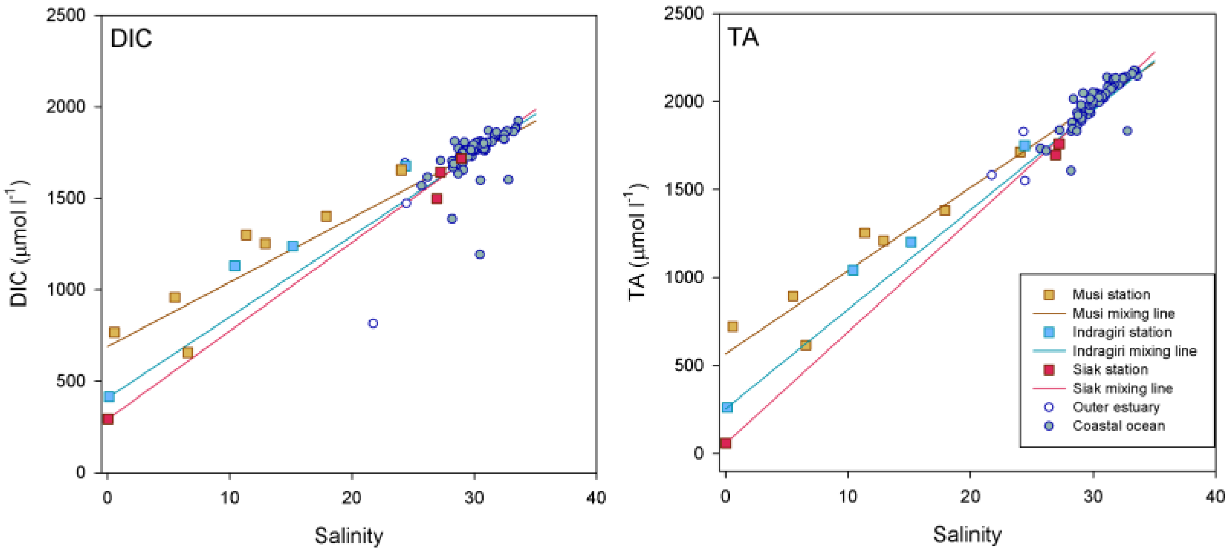

Supplementary Figure 3: Mixing lines for DIC (a) and TA (b) for the Musi, Indragiri and Siak rivers, as well as the average mixing line for Sumatra.

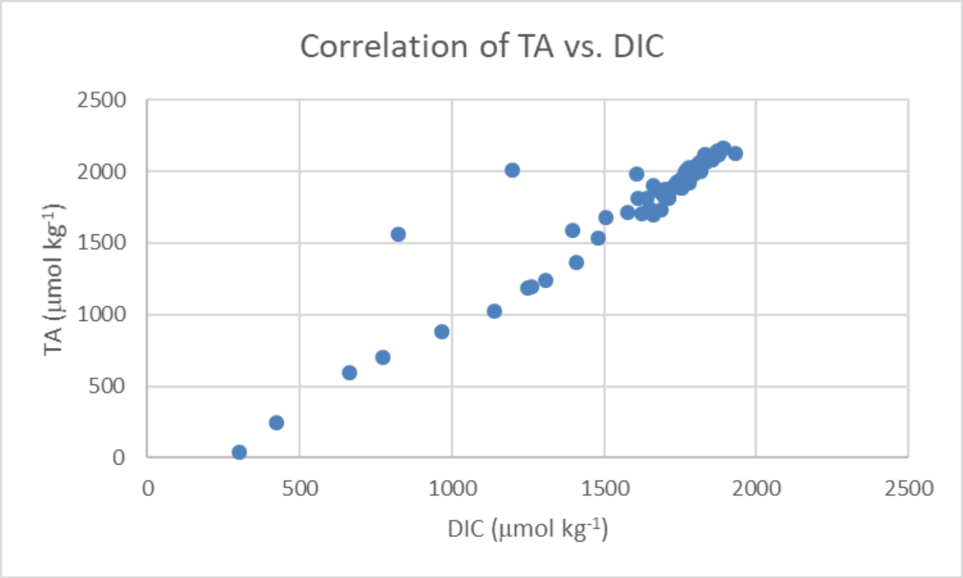

Supplementary Figure 4: Correlation between the TA and DIC concentrations in µmol kg-1.

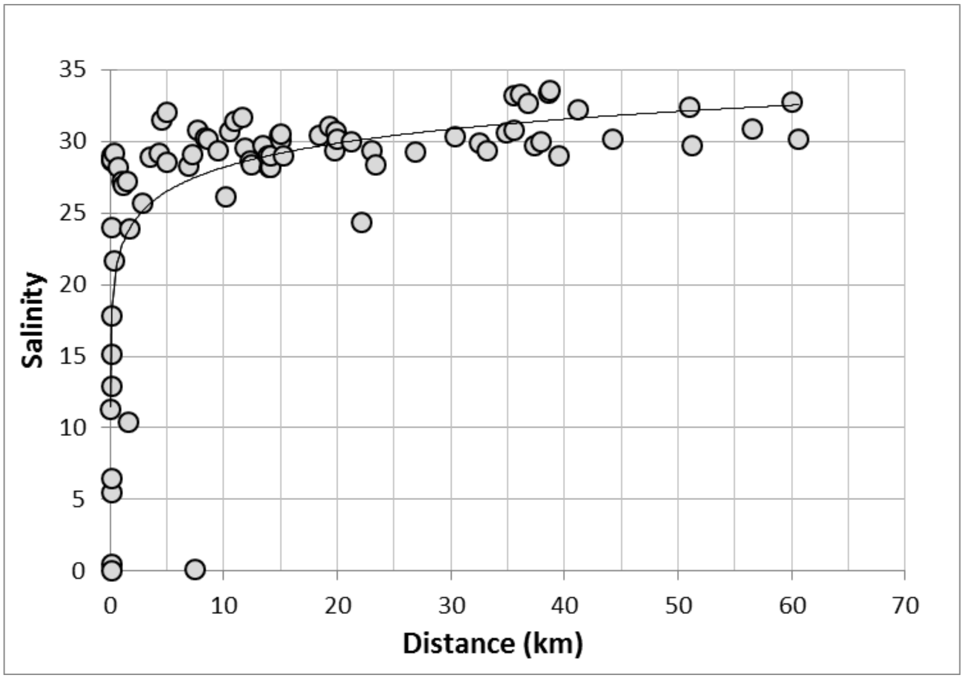

Supplementary Figure 5: Correlation salinity and distance from shore. Salinity 25, which is considered the border between the estuaries and coastal ocean, is located at approximately 3 km distance. The border between the coastal ocean and open ocean is located at circa 67 km distance.

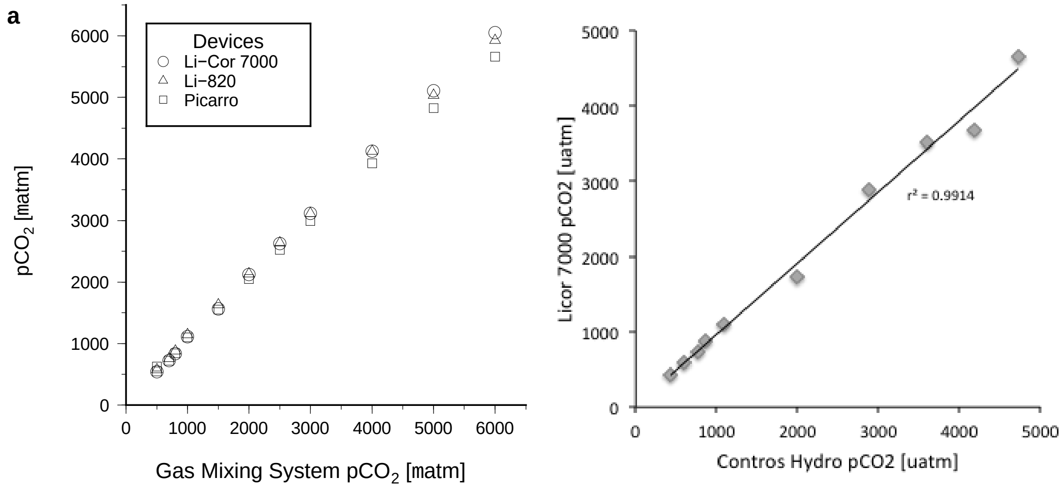

Supplementary Figure 6: a) Monitor results of calibration gas using the mixing system, Li-Cor 7000, Li-820 and Picarro devices. b) Calibration experiment results, $r^2 = 0.99$

Supplementary Table 1: Precipitation, discharge and end-member concentrations of DOC, DIC, POC and PIC for the rivers. Uncertainties of the values per expedition are the standard deviation, whereas uncertainties of the averages are the standard error.

River	Expedition	Precipitation mm	Discharge $m^3 s^{-1}$	DOC $\mu mol\ l^{-1}$	DIC $\mu mol\ l^{-1}$	POC $\mu mol\ l^{-1}$	PIC $\mu mol\ l^{-1}$
Musi	Mar 2008	352	4735	/	/	117	/
	Nov 2008	436	5865	423±21	/	242	/
	Oct 2009	197	2650	223±5	/	70	8
	Oct 2012	249	3350	264±5	748±42	/	/
	Apr 2013	238	3202	/	/	/	/
	**Average**	**294±44**	**3961±587**	**303±61**	**748±42**	**143±51**	**8±-**
Batanghari	Oct 2009	214	2270	377±10	/	109	10
	Oct 2012	249	2641	241±1	/	/	/
	Apr 2013	190	2015	314±0	/	/	/
	**Average**	**218±17**	**2309±182**	**311±39**	**/**	**109±-**	**10±-**
Indragiri	Mar 2008	366	1554	846±159	/	449	/
	Nov 2008	291	1236	/	/	304	/
	Oct 2009	272	1155	774±71	/	692	57
	Apr 2013	332	1410	651±5	409±12	/	/
	**Average**	**315±21**	**1339±89**	**757±57**	**409±12**	**482±113**	**57±-**
Kampar	Mar 2006	290	1795	1236±51	/	103	/
	Mar 2008	429	2655	/	/	133	/
	Nov 2008	281	1739	1325±39	/	232	/
	**Average**	**333±51**	**2063±297**	**1280±45**	**/**	**156±39**	**/**
Siak	Sep 2004	250	616	2187±40	/	492	/
	Aug 2005	426	1049	2159±136	/	836	/
	Mar 2006	216	532	1633±55	/	316	/
	Nov 2006	244	601	1849±22	/	/	/
	Mar 2008	409	1007	2205±56	/	443	/
	Nov 2008	273	672	/	/	135	/
	Oct 2009	317	781	2632±164	/	773	0.00
	Apr 2013	205	505	633±88	291±11	/	/
	**Average**	**293±46**	**720±74**	**1900±242**	**291±11**	**499±109**	**0.00±-**
Rokan	Apr 2006	300	1365	833±50	/	/	/
	Mar 2008	438	1993	728±54	/	1017	/
	Nov 2008	255	1160	/	/	1052	/
	**Average**	**331±67**	**1506±307**	**781±53**	**/**	**1034±18**	**/**

Supplementary Table 2: CO_2 outgassing fluxes based on Nightingale's principle for K of the rivers, estuaries and coastal ocean of Sumatra based on averaged concentrations measured during expeditions from 2009 to 2013. Errors are represented as the standard error.

Location		Estuaries	Coastal ocean	Subtotal marine
Area	km^2	10818	127674	138492
Wind speed	m/s	5.59±0.41	5.59±0.41	/
K_N	$cm\ hr^{-1}$	10.9±1.4	10.9±1.4	/

pCO_2 conc.	µatm	2038±56	554±1	/
CO_2 yield	g C m^{-2} yr^{-1}	609.2±79.9	44.9±5.9	/
CO_2 flux	Tg yr^{-1}	6.6±0.9	5.7±0.8	12.3±1.6

K is based on Nightingale. The spread of the K_N, CO_2 yields and fluxes are best/worst case scenarios, calculated based on the s.d. of the wind speed. The spread of the pCO_2 is the s.e.

Supplementary Table 3: Measurements station data

Station	Expedition	Latitude	Longitude	T [°C]	S -	pH -	pCO_2 [µatm]	TA [µmol kg $^{-1}$]
1	Oct.2009	0.41450	103.63483	29.38	28.75	7.94	507	1870
2	Oct.2009	0.56817	103.32250	29.42	24.44	7.76	796	1551
3	Oct.2009	1.71050	102.23133	29.90	29.06	7.86	483	1892
4	Oct.2009	1.70733	101.89867	25.85	24.32	7.86	534	1830
5	Oct.2009	1.66367	101.62250	30.10	28.20	7.80	733	1833
6	Oct.2009	1.90900	101.36650	30.54	29.20	7.84	538	1930
7	Oct.2009	2.34183	101.13550	30.32	29.40	7.82	475	1963
8	Oct.2009	2.49683	100.80400	30.05	29.54	7.87	518	1925
9	Oct.2009	2.35950	100.52183	29.81	29.69	7.91	566	1950
10	Oct.2009	2.66917	100.46517	31.47	30.66	7.95	410	2039
11	Oct.2009	2.91717	100.46450	31.21	30.82	7.94	432	2017
12	Oct.2009	3.33433	100.33033	30.54	30.94	8.00	431	2022
13	Oct.2009	2.65933	101.03217	30.45	29.81	7.95	514	1936
14	Oct.2009	2.42117	101.33300	30.82	29.45	7.89	486	1951
15	Oct.2009	2.36183	101.58633	30.12	29.30	7.88	536	1952
16	Oct.2009	2.16600	101.82600	30.43	29.00	7.85	539	1898
17	Oct.2009	1.69733	102.52350	30.01	29.33	7.99	517	1964
18	Oct.2009	0.86267	103.58050	29.82	29.04	7.83	648	1901
19	Oct.2009	-1.57900	105.18267	30.93	32.80	7.94	431	1834
20	Oct.2012	-5.94378	106.10849	30.91	33.59	7.86	626	2146
21	Oct.2012	-5.80374	106.15040	29.99	33.44	7.97	430	2176
22	Oct.2012	-5.58243	106.15214	30.51	33.35	7.97	432	2178
23	Oct.2012	-5.38346	106.15762	30.67	33.20	7.96	427	2158
24	Oct.2012	-4.00969	106.25901	29.65	32.67	7.97	447	2140
25	Oct.2012	-3.62071	106.28497	30.29	32.20	7.98	445	2103
26	Oct.2012	-2.41814	105.68507	29.78	32.08	7.93	474	2097
27	Oct.2012	-2.29437	105.67489	30.13	31.71	7.96	450	2098
28	Oct.2012	-2.35270	105.63456	30.01	31.56	7.94	457	2081
29	Oct.2012	-2.17715	104.98203	29.76	30.25	7.97	414	2050
30	Oct.2012	-2.38157	104.91312	28.90	11.36	7.19	2237	1254
31	Oct.2012	-2.49362	104.94101	29.75	5.51	7.07	2399	894
32	Oct.2012	-2.76004	104.95005	30.85	0.57	7.17	1716	722
33	Oct.2012	-2.55027	104.93951	30.83	12.90	7.25	2298	1210

34	Oct.2012	-2.47059	104.93963	30.86	17.89	7.40	1943		1382
35	Oct.2012	-2.37509	104.91016	30.55	24.05	7.67	1111		1714
36	Oct.2012	-2.32164	104.91951	30.50	28.69	7.90	641		1937
37	Oct.2012	-2.08435	104.99612	30.80	31.45	8.01	441		2071
38	Oct.2012	-1.65522	105.14807	30.66	32.45	8.01	425		2136
39	Oct.2012	-1.21777	105.21603	31.66	32.45	8.02	384		2137
40	Oct.2012	-1.14342	105.26736	30.78	32.44	8.02	381		2133
41	Oct.2012	-0.93883	104.08429	30.75	30.80	8.02	433		2028
42	Apr.2013	-2.29195	104.92429	28.94	6.56	6.84	1594		616
43	Apr.2013	-2.00165	104.93436	31.84	29.11	7.96	370		1917
44	Apr.2013	-1.79055	104.71477	33.48	28.26	7.88	499		1881
45	Apr.2013	-1.13757	104.53207	31.06	29.01	7.91	468		1980
46	Apr.2013	-1.00038	104.34203	30.23	25.69	7.82	482		1734
47	Apr.2013	-1.00001	103.80985	32.01	21.73	7.55	690		1583
48	Apr.2013	-0.98328	103.80527	29.45	27.25	7.69	727		1839
49	Apr.2013	-0.71203	103.73110	30.66	24.40	7.68	1099		1751
50	Apr.2013	-0.47392	103.66599	32.45	0.14	6.27	5466		262
51	Apr.2013	-0.19012	103.58838	31.34	10.42	6.99	4019		1043
52	Apr.2013	-0.12062	103.56937	31.54	15.15	7.30	2459		1202
53	Apr.2013	0.19504	103.48306	29.83	30.20	7.88	536		1998
54	Apr.2013	0.34466	103.44214	30.24	31.05	7.91	499		2044
55	Apr.2013	0.51584	103.39533	31.09	29.04	7.78	685		1903
56	Apr.2013	0.61141	103.36920	31.32	28.32	7.75	674		1846
57	Apr.2013	0.74301	103.33321	30.68	26.17	7.62	851		1721
58	Apr.2013	0.93607	103.28042	29.67	28.19	7.70	729		1608
59	Apr.2013	1.17703	103.09466	30.12	30.50	7.88	542		1999
60	Apr.2013	1.28996	102.79497	30.34	29.72	7.85	559		1981
61	Apr.2013	1.52619	102.58410	30.38	30.76	7.89	533		2027
62	Apr.2013	1.70728	102.23696	30.23	30.47	7.84	515		2023
63	Apr.2013	1.37205	102.16746	30.08	26.94	7.84	772		1698
64	Apr.2013	1.00022	102.10861	30.11	0.04	5.56	7747		60
65	Apr.2013	1.38722	102.15991	29.85	27.22	7.69	782		1759
66	Apr.2013	1.53412	101.99039	31.86	28.94	7.84	616		1905
67	Apr.2013	1.90886	101.37131	30.07	28.76	7.95	549		1921
68	Apr.2013	2.50031	100.80542	30.67	28.39	7.88	564		2015
69	Apr.2013	2.36372	100.52396	31.24	28.68	7.89	505		1833
70	Apr.2013	2.66175	100.47260	30.38	30.16	7.97	438		2018
71	Apr.2013	2.91266	100.46034	30.39	30.64	7.98	415		2042
72	Apr.2013	2.04896	101.35502	29.60	29.19	7.95	555		2048
73	Apr.2013	2.95053	100.71134	30.10	30.17	7.89	545		2002
74	Apr.2013	2.66434	101.02469	30.94	30.04	7.89	528		2000
75	Apr.2013	2.42507	101.32436	31.12	29.93	7.93	505		1987
76	Apr.2013	2.31455	101.61946	31.35	29.98	7.93	515		2000

77	Apr.2013	2.16576	101.83574	30.73	30.08	7.91	525	2014
78	Apr.2013	1.68694	102.51733	30.37	30.49	7.93	513	2043
79	Apr.2013	1.25835	103.20830	31.62	30.32	7.90	538	2041
80	Apr.2013	1.11693	103.49445	31.47	30.15	7.90	541	2027
81	Apr.2013	1.00013	104.70169	30.04	31.14	7.93	461	2138
82	Apr.2013	1.00013	104.70169	31.13	31.19	7.93	427	2084
83	Apr.2013	-0.01471	105.02102	30.53	31.65	7.97	465	2130
84	Apr.2013	-0.82931	105.27735	30.16	31.82	7.97	462	2134
85	Apr.2013	-1.20290	105.32004	30.83	29.95	7.99	449	2052
86	Apr.2013	-1.65526	105.15197	30.89	29.73	7.99	447	2015
